# Faecopneumothorax Caused by Perforated Diaphragmatic Hernia

**DOI:** 10.1155/2020/8860336

**Published:** 2020-08-10

**Authors:** Kristina Necke, Nickolaus Heeren, Francesco Mongelli, Maurice FitzGerald, Jürgen Fornaro, Fabrizio Minervini, Jürg Metzger, Jörn-Markus Gass

**Affiliations:** ^1^Department of Surgery, Cantonal Hospital of Lucerne, Lucerne, Switzerland; ^2^Department of Surgery, Regional Hospital of Bellinzona, Bellinzona, Switzerland; ^3^Department of Radiology, Cantonal Hospital of Lucerne, Lucerne, Switzerland; ^4^Department of Thoracic Surgery, Cantonal Hospital of Lucerne, Lucerne, Switzerland

## Abstract

Incarcerated diaphragmatic hernias with intrathoracic perforation of the colon is a very rare but serious surgical emergency. A 78-year-old male patient presented to our emergency department with severe abdominal pain. A computer tomography (CT) scan revealed herniation of the left transverse colon and spleen into the thorax with colon perforation and fecal contents in the thoracic cavity. An emergent laparotomy confirmed the radiological diagnosis and showed a 6 cm dehiscence of the left diaphragm with strangulation of the left transverse colon as well as the spleen. A left-sided hemicolectomy with terminal transversostomy and splenectomy were performed. The diaphragm was closed with interrupted nonabsorbable sutures. We abstained from reinforcement of the suture line with a mesh because of the feculent contamination of the abdominal cavity. After extensive thoracoscopic lavage and insertion of two chest tubes, the patient was transferred to the intensive care unit. Diaphragmatic hernia even after a mild chest trauma can cause fatal complications. Diagnosis and treatment can be challenging and an interdisciplinary approach is recommended. Due to the associated comorbidity and long-lasting sequelae, we believe the awareness of this rare pathology as a differential diagnosis is important; both as an abdominal and thoracic emergency.

## 1. Introduction

The incidence of diaphragmatic injuries after blunt or penetrating chest and abdominal trauma is1.1-3% [1]. In the emergency setting, diaphragmatic injuries can be overseen and the real overall incidence is probably underestimated. In blunt trauma, a sudden rise of intraabdominal pressure can lead to diaphragmatic rupture as in our case. Left-sided ruptures are more frequent because of the protective effects of the liver (71% vs. 29%) [2, 3]. Clinical signs and symptoms of diaphragmatic rupture after mild chest trauma are usually nonspecific and heterogenic [4], and on first admission, the diagnosis may be missed [2, 5]. Complications like bowel obstruction with strangulation or perforation can occur much later and have a very high mortality rate of up to 25% [6]. Therefore, in patients with a history of any kind of chest trauma presenting with unspecific symptoms in the emergency department, a thoracoabdominal CT scan should be performed to rule out diaphragmatic hernia. We present a case of herniation of intestinal organs with perforation of the incarcerated left transverse colon and a review of the literature.

## 2. Case History/Clinical Examination

A 78-year-old male patient presented with acute onset of severe left upper quadrant pain, hypotension, and dyspnea in our emergency department. On medical history, the patient was treated conservatively for left-sided rib fractures VII-XI after a moderate chest trauma 6 months prior to the presentation. Blood tests showed low white cell blood count (1.7 × 10^9^/L) indicating sepsis, C-reactive protein of 17 mg/L, and he was afebrile. As the patient was known for an abdominal aortic aneurysm 5.3 cm in diameter, a rupture was the first suspicion.

Clinical examination showed diffuse pain on palpation and local signs of peritonism in the left upper quadrant. Bowel sounds were rare but of normal quality. Heart rate was within normal limits at 80 beats per minute, and he was hyptotensive (80/60 mg/Hg). Oxygen saturation was 90% without supplementation.

### 2.1. Differential Diagnosis, Investigations, and Treatment

**Figure 1:**
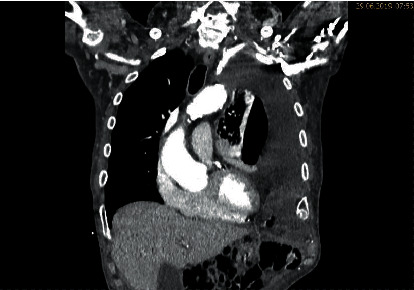
Coronary thoracoabdominal CT scan with left-sided seropneumothorax.

**Figure 2:**
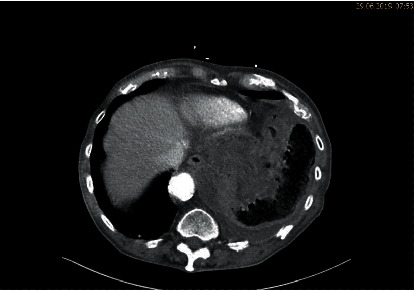
Axial thoracoabdominal CT scan with left-sided seropneumothorax.

A thoracoabdominal CT scan was performed identifying a left-sided diaphragmatic hernia with perforation of the transverse colon and feculent liquid and free air in the left thoracic cavity with right-sided mediastinal shift (Figures 1 and 2).

**Figure 3:**
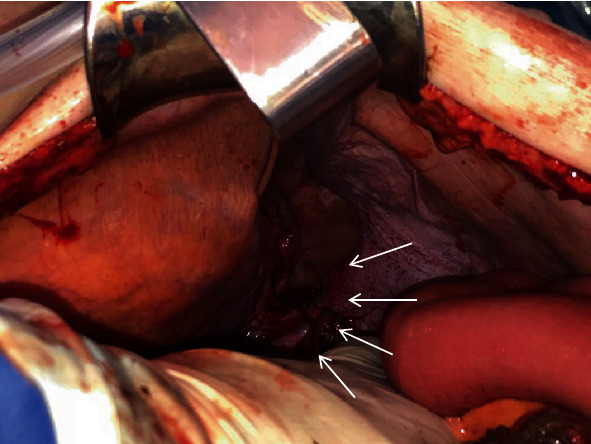
Intraoperative picture. Arrows indicating diaphragmatic defect.

Due to the unstable condition, we proceeded with an emergency laparotomy. Intraoperative findings showed a 6 cm diaphragmatic hernia with herniated contents including a perforated left splenic flexure of the transverse and left colon and partial herniation of the spleen (Figure 3). The abdominal cavity showed a feculent peritonitis, and the pleural cavity was contaminated with liquid feces. The herniated organs were reduced into the abdominal cavity and a left hemicolectomy and splenectomy performed as a damage control procedure. After an extensive saline lavage of the abdominal and thoracic cavity through the diaphragmatic hernia, the defect was closed with interrupted nonabsorbable sutures. In this hemodynamic unstable patient, we decided to perform a discontinuity resection with terminal colostomy of the transverse colon. Because of the bacterial contamination, the diaphragmatic defect was not reinforced by a mesh. A thorough lavage of the thoracic cavity was performed via the diaphragm and completed by thoracoscopy with early decortication and insertion of an apical and basal intercostal chest drain.

### 2.2. Outcome and Follow-Up

The patient had to stay in the intensive care unit for 6 days and was discharged after an uneventful recovery on the 20th postoperative day. During his last visit 9 months after the procedure, the patient was asymptomatic and had return to previous activity levels.

## 3. Discussion

Posttraumatic diaphragmatic hernias are rare but well-known complications after blunt (1-7%) or penetrating (10-15%) thoracic or abdominal trauma. They can occur even after a mild to moderate chest trauma. The pathomechanism of a diaphragmatic rupture after blunt abdominal trauma is a sudden increase of intra-abdominal pressure, e.g., after high energy acceleration-deceleration trauma. The mechanism in penetrating trauma is a direct injury caused by direct sharp dissective trauma as in gunshot or in knife wounds [7]. Left-sided injuries are more frequent than right-sided probably because of the protective effect of the liver and increased strength of the right part of the diaphragm [8, 9].

Symptoms can initially be masked by more acute life-threatening injuries (aorta, kidney, hollow viscera, liver, lung, spleen, pelvic, and rib fractures). Patients can present symptoms immediately after the trauma or months and years later and therefore, a direct correlation with the initial trauma is not always obvious [10, 11]. Moreover, most patients with delayed presentation have nonspecific symptoms and thus the diagnosis can be missed [12, 13]. Respiratory distress is the most common symptom on presentation due to herniation of the viscerous organs into the thoracic cavity. Some patients have long symptom-free intervals and suddenly develop substantial complaints because of complications such as obstruction, strangulation, or perforation [13–15].

Grimes described in 1974 the three phases of diaphragmatic rupture. Patients in the acute phase are diagnosed more easily because the correlation between the initial trauma and the rupture is more obvious. In the latent phase, patients can be asymptomatic over years and sometimes the diagnosis is only made coincidentally. The obstructive phase is characterised by a latency period between the trauma and pathological progression and onset of symptoms [16].

With a high index of suspicion in patients with a history of any kind of chest or abdominal trauma, a CT scan can help to assess the right diagnosis. Compared to a chest X-ray with a sensitivity of 17-48%, the CT scan can confirm the correct diagnosis in 71-100% [7, 9, 17]. The diagnosis of traumatic diaphragmatic injuries can be challenging and is initially often missed (12-66%). Over the last years, CT scan is widely used for patients with chest or abdominal trauma. Sensitivity is almost 100% and typical signs of the CT scan are herniation of visceral organs, direct visualization of the diaphragmatic defect, the so called “collar sign,” and partial nonvisualization of diaphragmatic integrity [18, 19].

In our case, the patient presented 6 months after the initial trauma with severe pain and respiratory distress due to a herniation and perforated incarceration of the colon into the left thoracic cavity leading to a faecopneumothorax with mediastinal shift to the right side. Faecopneumothorax is an extremely rare but serious presentation after blunt thoracoabdominal injury and traumatic diaphragmatic hernia with only very few cases published in literature to date [13].

Chern et al. reported in 2018 a case of a 22-year-old patient who presented repeatedly at the emergency ward with a history of left upper quadrant and chest pain. Additionally, he complained of nausea, vomiting, and intermittent constipation. At the final presentation at the emergency department, he was septic with tachycardia and hypotension. Two years ago, he was stabbed into the left lower chest. Intraoperatively, a 7-10 cm diaphragmatic hernia was found. The splenic flexure of the left colon showed a 3 mm perforation and caused intrathoracic and intraabdominal contamination. In contrast to our much older patient, a segmental resection with primary anastomosis was performed. The diaphragm was reinforced with a biosynthetic mesh [13].

Khan et al. presented in 2009 the case of a 32-year-old male patient with respiratory distress 5 years after a penetrating left thoracic stab wound. Intraoperatively, a 7 × 2 cm defect of the left diaphragm was visible and transverse colon was incarcerated and perforated. As in our patient, a Hartmann's procedure was performed and the defect was closed anatomically without implantation of a mesh [20].

According to literature, the closure of the defect can be done by a running suture or interrupted sutures [12]. Obviously, both techniques are equally effective. A nonabsorbable suture is recommended as well as the implantation of a synthetic mesh in the uncontaminated situation.

Routine access for diaphragmatic hernias is laparotomy or sometimes thoracotomy. In selected cases and experienced hands, an endoscopic approach can be feasible [21]. Most authors recommend laparotomy for acute traumatic rupture of the diaphragm or for complicated situations like incarceration and strangulation [17]. This offers the possibility to investigate and explore all the visceral organs more thoroughly than via laparoscopy and to exclude concomitant injuries.

## 4. Conclusion

Delayed complications after mild chest trauma with posttraumatic diaphragmatic hernias are a diagnostic challenge and can be in threatening conditions. Timely and accurate diagnosis and a prompt surgical intervention are key points to minimize morbidity and mortality. In our experience, a combined abdominal and a minimally invasive thoracic approach is advised if a perforation in the thoracic cavity is already present. Even after a minor thoracic trauma in the past, complications of a diaphragmatic hernia should be taken into account and a high index of suspicion should lead to the correct diagnosis. Reinforcement with a composite mesh after direct closure of the diaphragm in a colonialized situation still remains under discussion.
